# Immune parameters in hospitalized COVID-19 patients during SARS-CoV2 delta and omicron waves

**DOI:** 10.1016/j.clinsp.2026.101213

**Published:** 2026-10-15

**Authors:** Geraldo S. Magalhães, Sarah N.C. Gimenes, Bianca C.L.F. Távora, José A. Portes Junior, Paula A.L. Calabria, Leonel I. Montealegre-Sanchez, Sandra C. Sampaio Vessoni, Eduardo Massad, Celina A. Lamas, Sergio S. Muller, Tania Regina Tozetto-Mendoza, Maria Cássia Mendes-Correa, Carlos R.R. Carvalho, Eliana L. Faquim-Mauro

**Affiliations:** a Immunopathology Lab, Butantan Institute, Sao Paulo, SP, Brazil; b Pulmonology Division, Institute of Heart, Hospital das Clínicas of the University of Sao Paulo Medical School (HCFMUSP) , Sao Paulo, SP, Brazil; c Digital Health, Technological Innovation Center (InovaHC), University of Sao Paulo Medical School (HCFMUSP), Sao Paulo, SP, Brazil; d Department of Infections and Tropical Disease, School of Medicine, University of Sao Paulo (FMUSP), Sao Paulo, SP, Brazil; e Virology Lab (LIM-52), Institute of Tropical Medicine, Medical School, University of Sao Paulo (FMUSP), Sao Paulo, SP, Brazil

**Keywords:** Sars-cov-2, Neutralizing antibodies, RNAemia, Cytokines, Clinical outcomes

## Abstract

Understanding immune mechanisms associated with COVID-19 severity remains important for interpreting clinical outcomes. We investigated virological and immunological profiles in 39-patients hospitalized with COVID-19 at InCor Hospital during the Delta and Omicron waves and included 28-non-hospitalized individuals with moderate COVID-19 as a comparator group for cytokine analyses. SARS-CoV-2 infection was confirmed by RT-PCR from nasopharyngeal swabs, and viral variants were identified by next-generation sequencing. RNAemia was assessed by RT-PCR and analyzed as a binary variable. Serum cytokines were quantified by cytometric bead array, while humoral responses were evaluated by anti-Spike RBD IgG levels using CLIA and a Wuhan-Hu-1–based pseudovirus neutralization assay. RNAemia was more frequent in ICU-admitted patients than in non-ICU patients, supporting an association with disease severity. Cytokine profiling showed increased IL-6, IL-8, TNF, IL-17 A and IL-10 levels in hospitalized patients compared with moderate COVID-19 individuals, but did not distinguish recovered from deceased patients. Neutralizing antibody titers measured against a Wuhan-Hu-1 spike pseudovirus were significantly higher in recovered individuals than decease patients, underscoring the relevance of antibody functionality. These data emphasize the relevance of viral dissemination and functional humoral immunity as potential biomarkers for COVID-19 progression and outcome, with RNAemia and neutralizing antibody levels showing a stronger exploratory association in this cohort.

## Highlights

• The vaccine impact on clinical outcomes in SARS-CoV-2 infection.
• High level of inflammatory cytokines is independently of clinical outcomes on viral infection.
• The presence of neutralizing antibodies are correlated with the recovery of SARS-CoV-2 infection.

## Introduction

The COVID-19 pandemic, caused by the Severe Acute Respiratory Syndrome Coronavirus-2 (SARS-CoV-2), has resulted in substantial morbidity and mortality worldwide. Since its emergence in late 2019, the virus has continued to evolve, giving rise to Variants Of Concern (VOCs) such as Alpha, Delta, and Omicron. These variants have contributed to successive infection waves with differing patterns of transmissibility, immune evasion, and clinical severity.^1^ As of early 2025, more than 6.3-million deaths have been reported globally, reinforcing the urgent need to understand the immune determinants of disease severity and recovery.

The clinical presentation of COVID-19 ranges from asymptomatic to critical illness. In severe cases, patients often experience an exaggerated inflammatory response, commonly referred to as a “cytokine storm”, which is characterized by elevated levels of pro-inflammatory cytokines such as IL-6, TNF-α, IL-1β, and IL-17A. This hyperinflammatory state is implicated in the development of Acute Respiratory Distress Syndrome (ARDS), multiorgan failure, and death.^2^, ^3^, ^4^

The humoral immune response also plays a critical role in the control of SARS-CoV-2 infection and several studies have shown that higher levels of neutralizing antibodies are associated with milder disease and better clinical outcomes.^5^, ^6^, ^7^ However, not all antibodies are neutralizing, and the presence of anti-SARS-CoV2 antibodies does not necessarily equate to protective immunity.

With the continued evolution of SARS-CoV-2, variant-specific mutations in the spike protein can affect viral fitness and immune recognition. The Delta variant was associated with increased disease severity and hospital burden, while Omicron, though generally causing less severe disease, demonstrated substantial immune escape against vaccine-induced and infection-induced antibodies.^8^^,^^9^

In Brazil, the period from September 2021 to February 2022 was marked by the circulation of both Delta and Omicron variants. Additionally, increasing attention has been given to RNAemia (detection of viral RNA in the bloodstream), as a potential marker of systemic viral dissemination and poor prognosis.^10^^,^^11^

In this context, we analyzed a single-center convenience cohort of 39 patients hospitalized with COVID-19 at InCor Hospital in São Paulo during the Delta and Omicron waves. Our study integrated viral sequencing, cytokine profiling, and humoral immune evaluation through both binding antibody quantification and pseudovirus-based neutralization assays. The pseudovirus assay, based on a replication-deficient SARS-CoV-2 pseudoparticle expressing the Wuhan-Hu-1 spike protein, allowed us to safely and effectively assess the functional neutralizing capacity of circulating antibodies in an antigenically standardized system.

Our findings revealed that cytokine levels measured at hospital admission were elevated in hospitalized patients compared with individuals with moderate COVID-19, but did not discriminate between clinical outcome groups. In contrast, RNAemia showed an association with disease severity, while higher neutralizing antibody titers were associated with a favorable clinical outcome. These data underscore the relevance of viral dissemination and functional humoral immunity in COVID-19 progression and outcome.

## Methods

### Study design and participants

This study included a single-center observational cohort of 39 patients hospitalized with confirmed COVID-19 at InCor Hospital, São Paulo, Brazil, between September 2021 and February 2022. This period encompassed the circulation of both the Delta and Omicron variants in Brazil. The study protocol was approved by the local Ethics Committee (approval number: CER 42,605,821.6.000.0068), and all patients or their legal representatives provided informed consent. The selection of the participants was performed by non-probability convenience sampling. Patients were invited to participate consecutively as they presented for hospital care. Those who met the eligibility criteria and provided written informed consent or whose legal representatives provided consent, were included. Because this was a convenience sample from a single tertiary hospital, the cohort may not represent the full clinical spectrum of hospitalized COVID-19 patients, and the generalizability of the findings is limited.

Inclusion criteria were hospitalization for COVID-19 and laboratory confirmation of SARS-CoV-2 infection by RT-PCR on nasopharyngeal swab samples collected at the time of hospital admission. The nasopharyngeal swab was collected and kept in saline buffer at 4 °C until the PCR and sequencing assay.

Blood samples were collected at the hospital admission for the antibody and cytokine profiles. Serum was obtained by blood centrifugation at 1500 rpm for 10-min at 20 °C. Serum samples were aliquoted and stored at −80 °C until use. Vaccination status was recorded for all participants at the time of hospital admission. Patients included individuals who were partially vaccinated (one or two doses), or fully vaccinated/boosted (three or more doses), according to the national vaccination program active during the study period. All vaccines administered were based on the ancestral (Wuhan-Hu-1) SARS-CoV-2 spike protein. The outcome variable comprised occurrence of death or recovery, that resulted in hospital discharge. Data for the other independent variables were obtained through retrospective review of medical records. As a comparator group for cytokine analysis, we included 28 serum samples from non-hospitalized individuals with PCR-confirmed SARS-CoV-2 infection and moderate symptoms, who had not been vaccinated. The exact timing of blood collection in relation to symptom onset was not uniformly available for this group.

This study was conducted and reported in accordance with the STROBE (Strengthening the Reporting of Observational Studies in Epidemiology) guidelines.

### RT-PCR and illumina sequencing for SARS-CoV-2 detection for variant identification and RNAemia evaluation

Nasopharyngeal swab samples were collected from all enrolled patients at the time of hospital admission for SARS-CoV-2 detection and viral variant identification. Total RNA was extracted using the PureLink™ Viral RNA/DNA Mini Kit (Invitrogen™, Thermo Fisher Scientific; Cat. n° 12,280,050), following the manufacturer’s protocol. SARS-CoV-2 infection was confirmed by Real-Time Reverse Transcription Polymerase Chain Reaction (RT-qPCR) targeting the spike gene. For viral lineage identification, RNA extracted from the swab samples was subjected to whole-genome sequencing using the Illumina platform. Libraries were prepared with a targeted amplicon approach, and sequencing was carried out according to the manufacturer’s instructions. Variant calling and lineage assignment were performed using established bioinformatic pipelines, including PANGOLIN.

RNAemia was assessed in serum samples collected at hospital admission. Viral RNA was extracted from the serum using the same PureLink™ Viral RNA/DNA Mini Kit, and SARS-CoV-2 RNA was detected by RT-qPCR targeting the spike gene. A positive serum RT-qPCR result was interpreted as detectable SARS-CoV-2 RNA in serum, not as evidence of infectious virus in blood. For statistical analyses, RNAemia was treated as a binary categorical variable, defined as detectable or non-detectable SARS-CoV-2 RNA in serum. Ct-values were not analyzed as continuous viral-load measurements.

### Cytokine quantification of Th1, Th2, and Th17 profiles by cytometric bead array (CBA)

Cytokine concentrations in serum samples from hospitalized patients and comparator volunteers were quantified using the BD™ Cytometric Bead Array (CBA) Human Th1/Th2/Th17 and Inflammatory Cytokine Profiles Kit (BD Biosciences, CA, USA), according to the manufacturer’s instructions. Briefly, serum samples from patients were incubated with a mixture of capture beads, each coated with a specific antibody for one of the target cytokines. A Phycoerythrin (PE)-conjugated detection antibody was then added to form sandwich complexes. Samples were subsequently analyzed on a BD FACSymphony™ A1 flow cytometer. Quantification of cytokine concentrations was performed by generating a standard curve with known concentrations of each cytokine, and data were analyzed using FlowJo™ v.10 software (BD Biosciences).

### Evaluation of anti-Spike RBD-Specific IgG by chemiluminescent immunoassay (CLIA)

The presence of IgG antibodies against the Receptor-Binding Domain (RBD) of the ancestral Wuhan-Hu-1 SARS-CoV-2 Spike (S) protein was evaluated in patient sera using a commercial Chemiluminescent Immunoassay (CLIA) kit (Elecsys® Anti-SARS-CoV-2 S, Roche Diagnostics, Basel, Switzerland), performed on the cobas® e analyzer platform, according to the manufacturer's instructions. This assay quantitatively detects antibodies directed against the RBD of the spike protein, a critical region involved in viral entry and a major target of neutralizing antibodies. Serum samples were processed using the double-antigen sandwich principle, in which recombinant RBD antigens are used to capture and detect specific IgG antibodies. The resulting chemiluminescent signal is proportional to the concentration of anti-RBD antibodies in the sample. Results were expressed in Binding Antibody Units per milliliter (BAU/mL), and values above the threshold recommended by the manufacturer were considered positive. All assays were performed under standardized laboratory conditions to ensure reliability and comparability across samples.

### Evaluation of serum neutralization by pseudovirus assay

To evaluate the neutralizing capacity of patient sera, we used a SARS-CoV-2 pseudovirus expressing the ancestral Wuhan-Hu-1 spike protein, corresponding to the antigen used in the RBD-based Chemiluminescent Immunoassay (CLIA). Serum samples from patients were serially diluted in three-fold steps, starting at a 1:10 dilution, in 96-well plates. Each diluted sample (60 µL) was incubated with a fixed amount of SARS-CoV-2 pseudovirus (60 µL) at a concentration of 4 × 10⁶ Relative Luminescence Units per milliliter (RLU/mL) for 1 hour at 37 °C. Following incubation, 100 µL of the virus-serum mixture was added to each well of a 96-well plate containing A549-hACE2-TMPRSS2 cells (10⁴ cells/well), previously seeded the day before in 100 µL of culture medium. After 24-hours of incubation at 37 °C, the culture supernatant was removed and the cells were lysed with 100 µL/well of One-Glo™ Luciferase Assay Reagent (Promega). Plates were incubated for 5-minutes at room temperature before measuring luminescence using a SpectraMax® M5 plate reader (Molecular Devices). Neutralization was calculated as the percentage of infection inhibition at each serum dilution relative to the virus-only control. A Four-Parameter Logistic (4PL) curve was fitted to the data to calculate the 50% Inhibitory Concentration (IC_50_) for each sample.

### Statistical analysis

For quantitative variables, mean, standard deviation, median, interquartile range, and minimum and maximum values were calculated. Categorical variables were described using absolute and relative frequencies. The distribution of quantitative variables was assessed using histograms and the Shapiro-Wilk normality test. As most variables showed non-normal distributions, nonparametric statistical methods were chosen for inferential analyses. For continuous variables the Mann-Whitney *U test* was performed for comparisons between two groups and the Kruskal-Wallis test followed by Dunn’s post-hoc test was used for comparisons among multiple groups, as appropriate.

In the bivariate analysis, associations between clinical variables and outcome (deceased or recovered) were evaluated using Fisher's Exact Test after construction of 2 × 2 contingency tables. RNAemia was also analyzed as a binary categorical variable, defined as detectable or non-detectable SARS-CoV-2 RNA in serum.

Because the cohort included only 11 deaths, multivariable logistic regression was not performed. The number of events was insufficient to support the adjustment for multiple predictors without substantial risk of overfitting, coefficient inflation, and unstable confidence intervals. Therefore, odds ratios, when reported, were restricted to exploratory bivariate estimates and should not be interpreted as independent effects. Results are presented as descriptive statistics, p-values, and, where applicable, unadjusted Odds Ratio (OR) and 95% Confidence Intervals (95% CI). Details of the statistical analysis are indicated in the corresponding figure legends and table footnotes. GraphPad Prism software version 11.0.2 was used for graph generation, normality assessment, Mann-Whitney *U tests*, Kruskal-Wallis tests, Dunn’s post-hoc tests, Fisher’s exact tests, contingency-table estimates and IC_50_ curve fitting. Statistical significance was defined as p < 0.05 (denoted as: ns, Non-Significant (p > 0.05); * p < 0.05; ** p < 0.01; *** p < 0.001; **** p < 0.0001).

## Results

### Participant characteristics and clinical outcomes

A total of 39 hospitalized patients were included, of whom 28 recovered and 11 deceased. Exploratory bivariate associations between clinical variables and clinical outcome (recovered or deceased) were analyzed using a 2 × 2 contingency table and Fisher’s Exact test (Table 1). The participants were categorized according to sex, age, vaccination, ICU admission status and the presence of comorbidities, moreover they were grouped as a clinical outcome (recovered or deceased). The proportion of female and male in each group was similar (around 50% in each group), and this parameter did not show a statistical difference, according to clinical outcome. Stroke, hypertension, obesity, lung disease, and the combined category of other comorbidities were also evaluated and did not differ significantly between recovered and deceased patients.

**Table 1:** Demographic and clinical characteristics at admission of hospitalized COVID-19 patients according to outcome (Recovered vs. Deceased). Bivariate association analysis was performed using the Fisher’s Exact test.

	Recovered		Deceased		Total		Fisher’s Exact Test	
	(n = 28)		(n = 11)		(n = 39)		p-value	OR (95% CI)
**Sex, n (%)**								
Female	14	(50%)	5	(45.45%)	19	(48.71%)	p = 1.0	1.194 (0.238‒6.235)
Male	14	(50%)	6	(54.54%)	20	(51.28%)		
**Age, n (%)**								
< 60-years	14	(50%)	1	(9.09%)	15	(38.46%)	c p *=* 0.028	9.501 (1.088‒463.11)
≥ 60-years	14	(50%)	10	(90.90%)	24	(61.53%)		
**Admission status, n (%)**								
ICU	8	(28.57%)	6	(54.54%)	14	(35.89%)	p = 0.155	0.344 (0.062‒1.784)
Non-ICU	20	(67.85%)	5	(45.45%)	25	(61.53%)		
**Vaccine Status, n (%)**								
One or two doses	9	(38.14%)	10	(90.90%)	19	(48.71%)	d p = 0.001	0.051 (0.001‒0.454)
Three or more doses	19	(67.85%)	1	(9.09%)	20	(51.28%)		
**Comorbidities, n (%)**								
Stroke before disease	2	(7.14%)	1	(9.09%)	3	(7.69%)	p = 1.0	0.775 (0.036‒49.869)
Heart disease a	4	(14.29%)	5	(36.36%)	9	(23.07%)	c p = 0.017	14.07 (1.166‒782.49)
Hypertension	1	(3.57%)	0	(0%)	1	(2.56%)	p = 0.482	1.825 (0.362‒9.672)
Obesity (BMI > 30)	7	(25.0%)	4	(36.36%)	11	(28.20%)	p = 0.694	0.592 (0.106‒3.611)
Lung disease	1	(3.57%)	1	(9.09%)	2	(5.12%)	p = 0.49	2.621 (0.031‒219.2)
Other b	9	(32.14%)	5	(45.45%)	14	(35.89%)	p = 0.649	2.668 (0.264‒137.7)

a Heart disease includes acute myocardial infarction, angina and heart failure.

b Other comorbidities include kidney disease, other chronic diseases, history of transplantation, immunosuppression, and/or depression. p-values calculated using Fisher's Exact Test. non-significant (p > 0.05).

c p < 0.05.

d p < 0.001.

Heart disease was the only comorbidity that differed significantly between outcome groups. A higher percentage of participants in the deceased group had heart disease before hospital admission (36.36%), whereas only 14.29% of participants in recovered group had reported heart disease before admission. These data indicate that heart disease was associated with mortality in this cohort in the bivariate analysis.

The age of participants also differed between outcome groups. The deceased group had a higher percentage of patients ≥ 60-years (90.90%) compared to the recovered group (50%). This finding suggests an association between older age and death in this cohort (Table 1). However, because of the small sample size and the limited number of deaths, these associations should be interpreted cautiously and should not be considered as an independent factor.

Vaccination status was another important variable that differed significantly between recovered and deceased patients. A higher percentage of participants in recovered group received three or more vaccine doses (67.85%) when compared with the deceased group (9.09%). These data suggest that receiving more vaccine doses was associated with recovery in the bivariate analysis. Conversely, one or two vaccine doses were more frequent among deceased patients.

This finding is consistent with a possible protective association of booster vaccination, but causality cannot be inferred from this observational convenience cohort because age, comorbidities, disease stage at admission, and other unmeasured factors may have influenced both vaccination status and outcome.

Because the cohort included only 11 deaths, no multivariable logistic regression model was retained in the revised analysis. This decision avoids overfitting and prevents overinterpretation of unstable adjusted odds ratios. Accordingly, clinical variables in Table 1 are presented as exploratory bivariate associations rather than independent predictors of mortality.

### Temporal distribution of SARS-CoV-2 variants and association of RNAemia with disease severity

To characterize the relationship between SARS-CoV-2 variant circulation and clinical outcomes, we first analyzed the temporal distribution of viral lineages among the 39 hospitalized patients. Viral RNA extracted from nasopharyngeal swab samples was sequenced using the Illumina platform, confirming SARS-CoV-2 positivity in all cases. From September to November 2021, Delta was the predominant variant, whereas from January to February 2022, Omicron became dominant (Fig. 1). A small proportion of cases across all months remained unclassified because of low coverage. These findings illustrate the temporal shift in variant circulation during the study period.

**Fig. 1:**
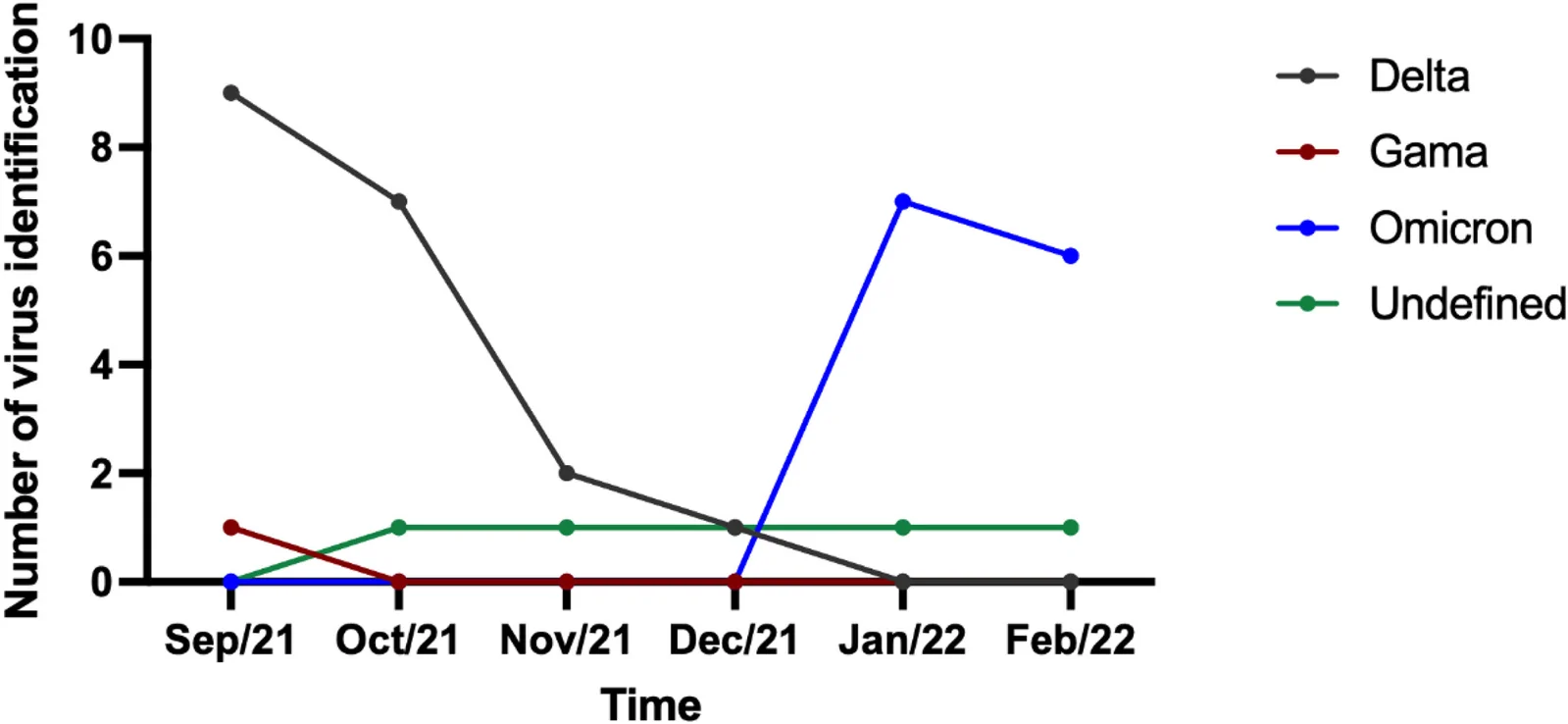
Temporal distributions of SARS-CoV-2 variants identified in 39 hospitalized patients at InCor Hospital (Brazil) between September 2021 and February 2022. Variants were assigned based on sequencing of viral RNA from nasopharyngeal swab samples. Samples with insufficient sequencing quality or coverage were classified as undefined.

In parallel, RNAemia was evaluated by RT-PCR in serum samples collected at hospital admission and analyzed as a binary variable, defined as detectable or non-detectable SARS-CoV-2 RNA in serum. A significantly higher frequency of RNAemia was observed among patients admitted to the Intensive Care Unit (ICU) compared with non-ICU patients. More than 70% of ICU patients showed detectable SARS-CoV-2 RNA in serum, whereas most non-ICU patients were negative for RNAemia (Fig. 2). These data indicate a marked difference in the frequency of RNAemia between ICU and non-ICU patients, and suggest that systemic viral dissemination is associated with greater disease severity. These findings suggest an association between detectable SARS-CoV-2 RNA in serum and ICU-level disease severity in this cohort. Because RNAemia was measured at hospital admission and analyzed only as a binary variable, these findings should not be interpreted as demonstrating that RNAemia is an independent factor of mortality.

**Fig. 2:**
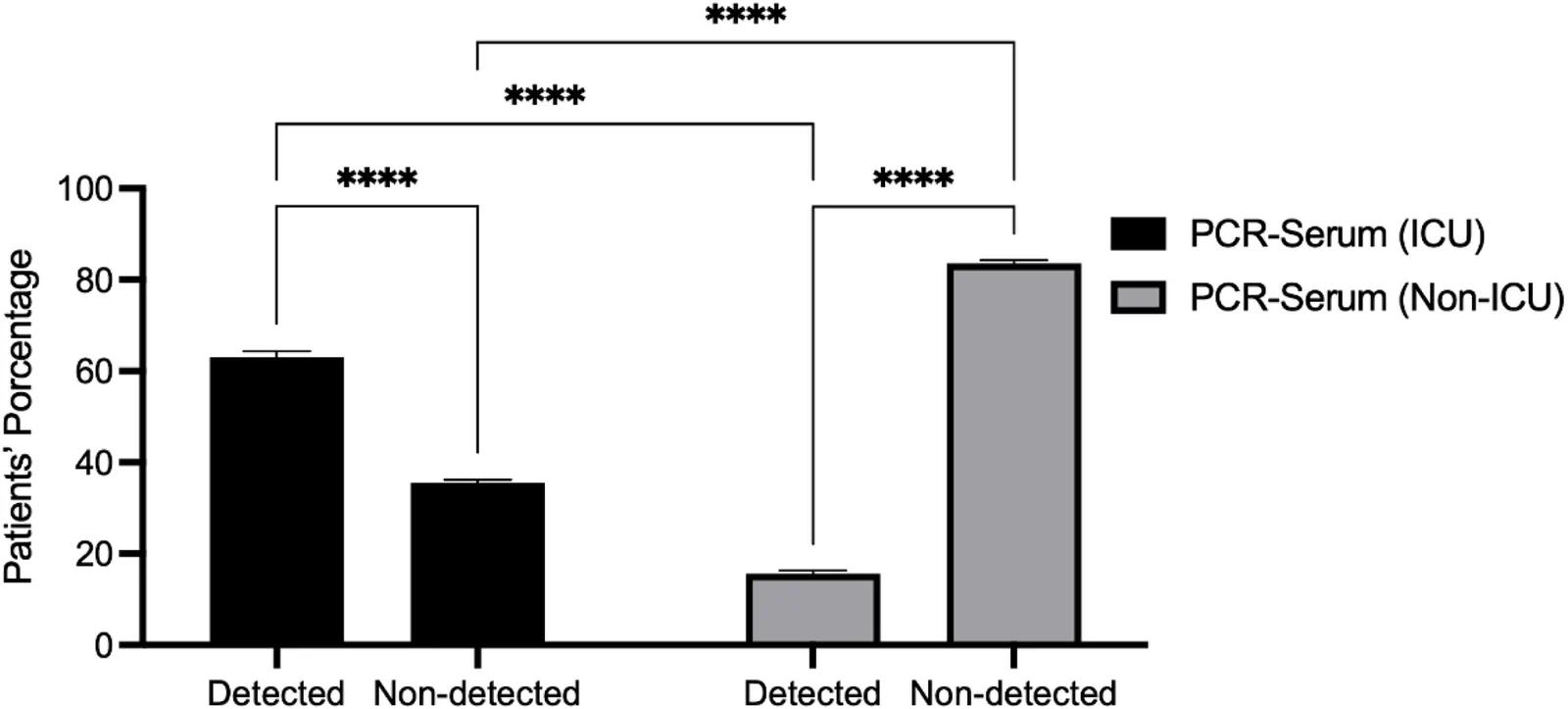
Association between RNAemia and clinical severity in hospitalized COVID-19 patients. Percentage of patients with detectable or non-detectable SARS-CoV-2 RNA in serum, stratified by ICU admission status. RNAemia was analyzed as a binary variable (detectable vs. non-detectable), and associations between groups were evaluated using Fisher’s exact test.; **** Indicates p < 0.0001.

### Pro-inflammatory cytokine profiles in recovered and deceased patients

To investigate the inflammatory response associated with COVID-19 severity, we measured serum levels of inflammatory and immunoregulatory cytokines in all participants at hospital admission and compared them with those of individuals with moderate COVID-19 who did not require hospitalization. A heatmap of individual cytokine concentrations demonstrated a broad distribution across recovered, deceased and moderate COVID-19 groups, with most patients displaying detectable levels of multiple pro-inflammatory cytokines (Fig. 3).

**Fig. 3:**
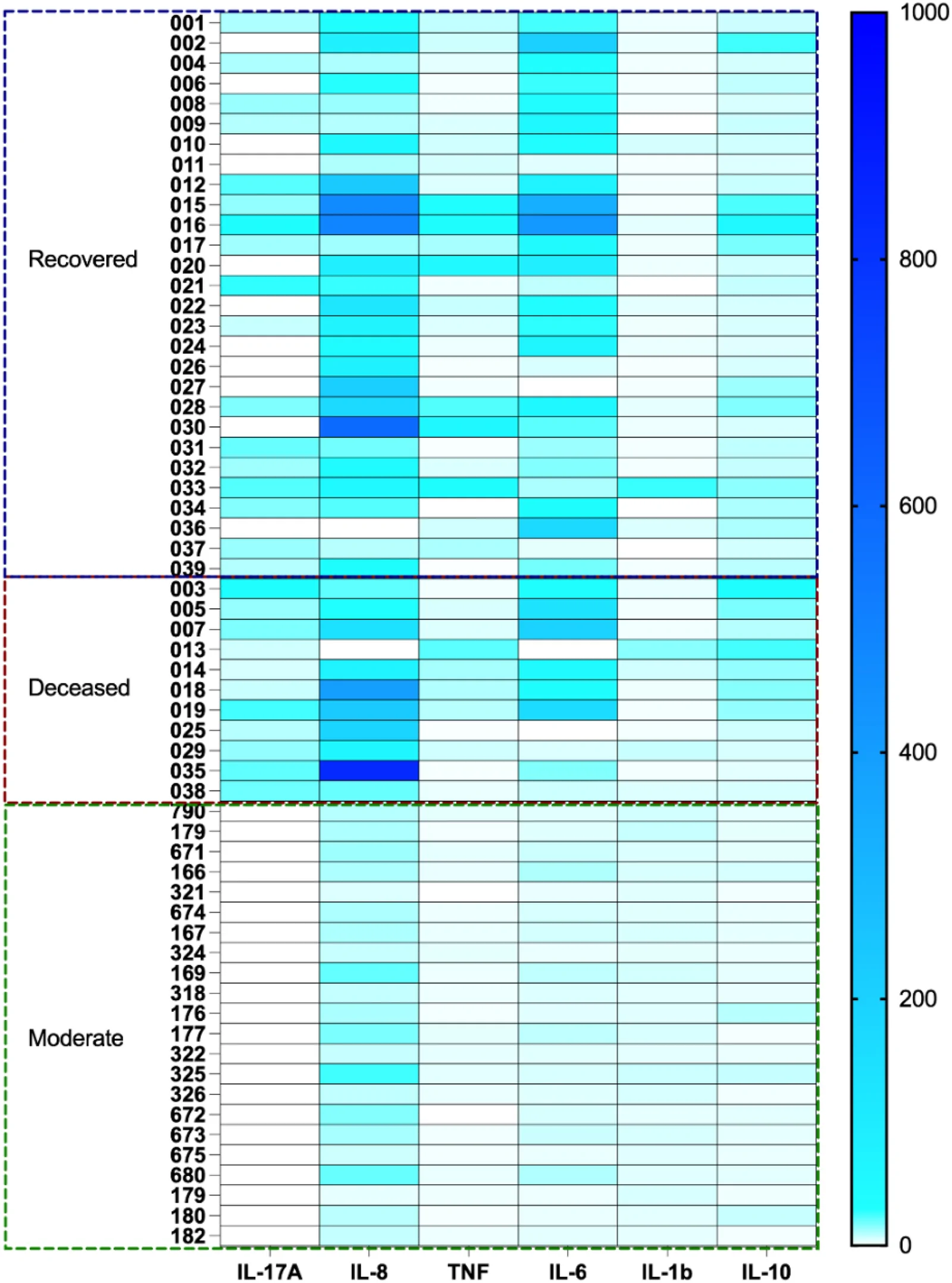
Heatmap of serum cytokine concentrations in hospitalized COVID-19 patients and individuals with moderate COVID-19 who did not require hospitalization. Production of IL-17A, IL-8, TNF, IL-6, IL-1β and IL-10 were measured in serum samples from 39 hospitalized patients categorized by clinical outcome (recovered or deceased) and 28 non-hospitalized individuals with moderate COVID-19 group. Each row represents an individual patient, and color intensity reflects the concentration of each cytokine (pg/mL), with a gradient scale ranging from low (light blue) to high (dark blue).

Our results also showed higher cytokine levels in hospitalized groups than in the moderate COVID-19 group, suggesting a more pronounced systemic inflammatory profile in all hospitalized patients. Therefore, in Fig. 4, we observed the elevated levels of IL-8, IL-6, in both groups of patients admitted to the hospital (recovered and deceased) when compared with the moderate-COVID-19 group. Furthermore, TNF-α, IL-17A and also anti-inflammatory IL-10 levels were higher in hospitalized groups compared with the moderate-COVID-19 group. However, despite this overall increase in inflammatory mediators and IL-10 among hospitalized patients, cytokine levels measured at hospital admission did not distinguish recovered from deceased outcomes.

**Fig. 4:**
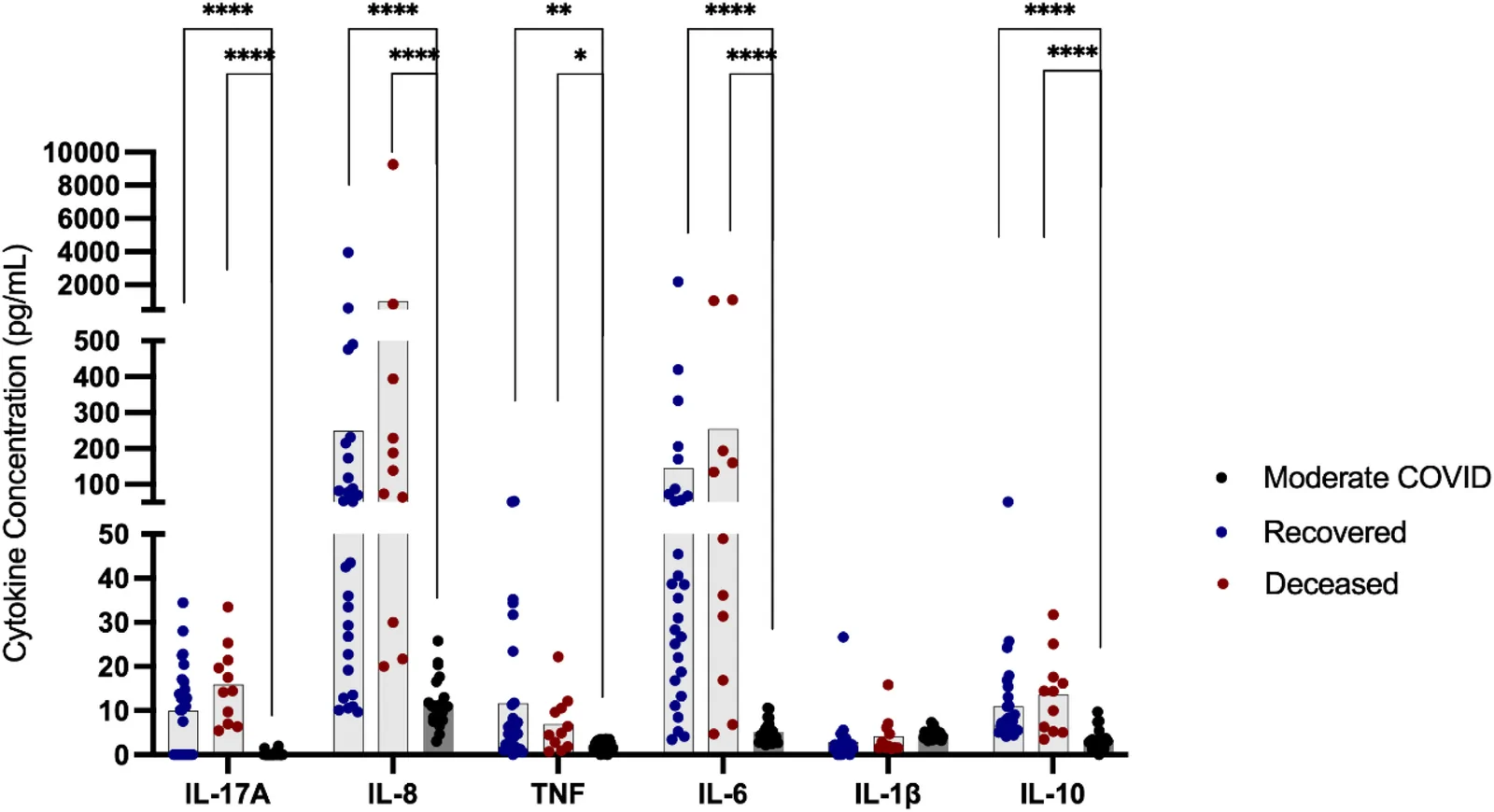
Comparative analysis of serum cytokine levels in hospitalized COVID-19 patients and individuals with moderate COVID-19. Serum concentrations of IL-17A, IL-8, TNF, IL-6, IL-1β, and IL-10 were quantified in 39 hospitalized patients (deceased and recovered) and compared with a group of 28 non-hospitalized individuals with moderate COVID-19. Each dot represents an individual patient: recovered (blue), deceased (red) and moderate COVID-19 infection (black). Kruskal-Wallis test followed by Dunn’s post-hoc test was used to compare the groups: moderate COVID, Recovered and Deceased. “ns” denotes non-significant comparisons. **** p < 0.0001, ** p < 0.01, * p < 0.05.

Taken together, our data suggest that a robust pro-inflammatory environment is a consistent feature of hospitalized COVID-19 patients, regardless of disease outcome. However, because cytokines are dynamic during infection and were assessed at a single time point, these findings should not be interpreted as excluding a prognostic role for longitudinal cytokine trajectories. Rather, they indicate that baseline cytokine concentrations at hospital admission were not sufficient to discriminate clinical outcome in this small cohort.

### Evaluation of spike-specific IgG binding and serum neutralization activity

To assess humoral immune responses against SARS-CoV-2 serum samples from hospitalized patients were analyzed using two complementary approaches: a chemiluminescent immunoassay for the detection of IgG antibodies specific to the Spike Receptor-Binding Domain (RBD), and a pseudovirus-based neutralization assay to evaluate the functional ability of serum antibodies to inhibit viral entry. Although patients in this cohort were infected during the Delta and Omicron waves, the humoral assays used here were designed to quantify antibody binding and functional neutralization against the ancestral Wuhan-Hu-1 spike/RBD, thereby providing a standardized readout of vaccine-antigen-matched and potentially cross-reactive humoral activity. Accordingly, the IC_50_ values reported below reflect neutralization of the ancestral spike and should be interpreted as a measure of cross-reactive functional antibody activity present in serum, rather than a direct measurement of neutralization against the infecting Delta/Omicron variant spike.

Quantification of anti-RBD antibodies revealed no statistically significant differences in antibody titers between recovered and deceased patients. Both groups exhibited a wide range of antibody concentrations, indicating heterogeneous humoral response regardless of clinical outcome (Fig. 5A). In contrast, exploratory non-parametric analysis showed that functional neutralization differed between the clinical groups. Recovered patients displayed significantly higher ancestral-spike neutralizing titers (IC_50_) than deceased patients (p < 0.01), suggesting that while total anti-RBD IgG levels were comparable, functional neutralizing activity was higher among recovered patients (Fig. 5B).

**Fig. 5:**
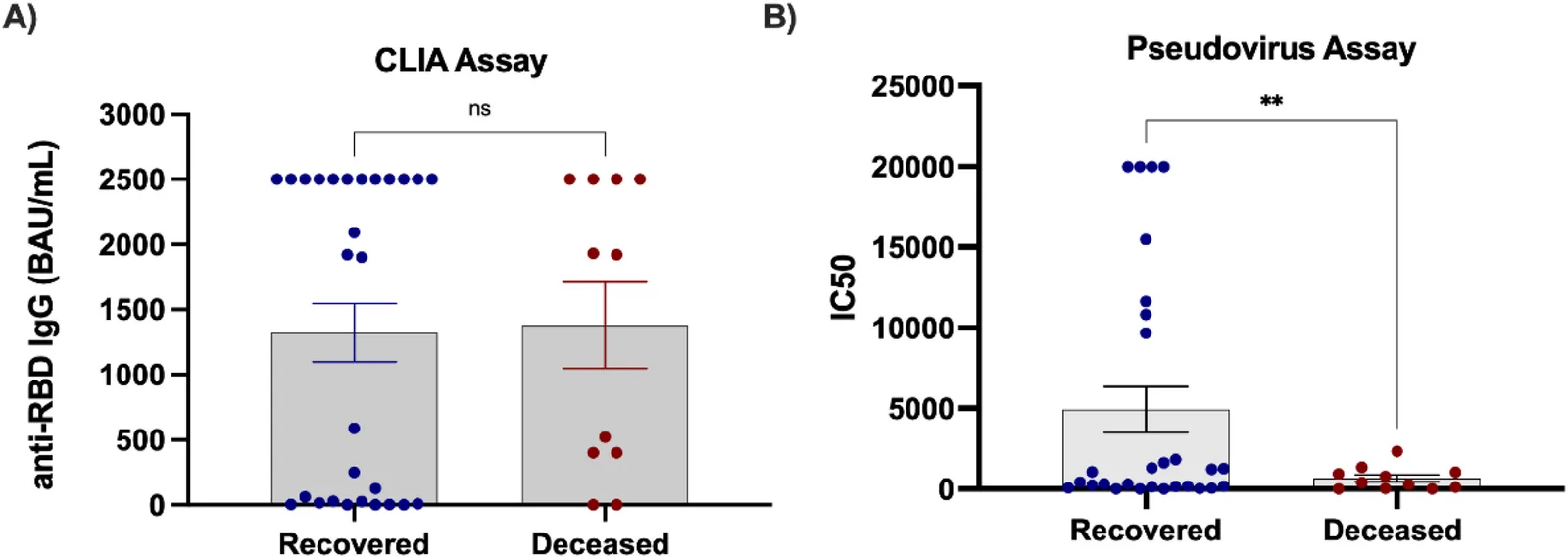
Evaluation of humoral immune responses against SARS-CoV-2 in hospitalized patients. (A) Detection of anti-RBD IgG antibodies in patient serum using a chemiluminescent immunoassay targeting the Receptor-Binding Domain (RBD) of the SARS-CoV-2 spike protein. No significant differences were observed between recovered and deceased groups. (B) Neutralizing activity of patient sera measured using a SARS-CoV-2 pseudovirus bearing the ancestral Wuhan-Hu-1 spike. Group comparisons in panels A and B were performed using the Mann-Whitney *U test* (two-tailed). Both the RBD-CLIA and the pseudovirus neutralization assay used the ancestral (Wuhan-Hu-1) spike/RBD antigens, providing an antigen-matched comparison between binding and neutralization measurements. ** p < 0.01; ns, non-significant.

Together, these findings suggest that functional neutralizing activity, rather than total anti-RBD binding antibody levels alone, was associated with recovery in this cohort. However, because the pseudovirus expressed the ancestral Wuhan-Hu-1 spike, this association should be interpreted as reflecting vaccine-antigen–matched and potentially cross-reactive neutralizing activity, not direct variant-specific neutralization of the infecting Delta or Omicron spike.

## Discussion

In this study, we evaluated virological, immunological and clinical parameters in a cohort of 39 hospitalized COVID-19 patients during the Delta and Omicron variant waves in Brazil. Our integrated approach aimed to identify exploratory associations between RNAemia, cytokine profiles, binding antibodies, functional neutralization, disease severity and clinical outcome. Because this was a single-center convenience cohort with only 11 deaths, the findings should be interpreted as hypothesis-generating and not as evidence of validated prognostic biomarkers or independent factor of mortality.

Regarding the clinical characteristics of this cohort, Table 1 showed that older age and heart disease, both more frequent among deceased patients. These variables were evaluated only in bivariate analyses, because the limited number of deaths did not support a reliable multivariable logistic regression model without a substantial risk of overfitting. Therefore, these clinical variables should be interpreted as unadjusted exploratory associations rather than independent predictors. This is consistent with previous evidence showing that older age and cardiovascular comorbidities increase vulnerability to severe COVID-19 and mortality.^12^

The analysis of RNAemia, detected by RT-PCR in serum, showed significantly more frequent RNAemia in ICU-admitted patients. This observation is consistent with studies by Bermejo-Martin et al. (2020) and Fajnzylber et al. (2020), which demonstrated that the presence of SARS-CoV-2 RNA in the bloodstream is associated with critical illness and higher mortality.^10^^,^^11^ RNAemia may reflect insufficient containment of viral replication in the respiratory tract, with systemic dissemination and immune overactivation. In our cohort, RNAemia was analyzed as a binary variable and was associated with ICU-level severity; therefore, it should not be interpreted as an independent signal of death, particularly because it was measured only at hospital admission and was not evaluated in an adequately powered multivariable mortality model.^13^^,^^14^

Although pro-inflammatory cytokines, such as IL-6, IL-8, IL-1β, TNF, and IL-17A were detected in both recovered and deceased patients, no statistically significant differences were observed between these groups when measured at hospital admission. However, with the inclusion of individuals with moderate COVID-19, who did not require hospitalization, cytokine levels were generally higher in hospitalized patients than in the moderate group. It is important to emphasize that from all groups presented, we could not control the time of sample collection and, therefore, our data represents a cross-sectional and may be affected by differences in sampling time. Among the cytokines analyzed, IL-6 and IL-8 were consistently elevated in sera from both outcome groups, reflecting a robust inflammatory response at the acute phase of infection. These findings are consistent with the known roles of IL-6 in systemic inflammation and IL-8 in neutrophil recruitment and lung inflammatory injury during severe COVID-19.^15^^,^^16^

Although IL-17A was included in the cytokine panel, its level did not show a statistically significant pattern supporting a specific Th17-driven interpretation in this cohort. Therefore, the cytokine profile observed here should be interpreted more broadly as evidence of systemic inflammation in hospitalized patients, rather than as a distinct Th17-associated signature.^15^^,^^16^ Increased circulating levels of pro-inflammatory cytokines have also been consistently associated with severe COVID-19 and Acute Respiratory Distress Syndrome (ARDS).^17^^,^^18^ In parallel, IL-10 was detected in both hospitalized outcome groups and has been described in severe SARS-CoV-2 infection, as part of a compensatory anti-inflammatory response aimed at limiting virus-induced hyperinflammation.^19^^,^^20^ Therefore, the concomitant detection of inflammatory cytokines and IL-10 in hospitalized patients suggests an active inflammatory response accompanied by compensatory immunoregulation. Because cytokine levels are dynamic over the course of infection, and the timing of blood collection relative to symptom onset was not uniformly available for the moderate COVID-19 comparator group, comparisons between hospitalized and moderate cases should be interpreted cautiously as cross-sectional rather than longitudinal. Accordingly, the absence of significant differences between recovered and deceased patients at admission should not be interpreted as excluding a prognostic role for longitudinal cytokine trajectories. Instead, our data indicate that baseline cytokine concentrations measured at a single time point were not sufficient to discriminate outcome in this small cohort.

Interestingly, the humoral immunity analysis showed that total anti-RBD IgG levels did not distinguish recovered from deceased patients. In contrast, recovered individuals exhibited significantly higher neutralizing antibody titers, measured using a Wuhan-Hu-1 – based pseudovirus. This ancestral-spike framework was chosen to align the neutralization assay with the RBD-based CLIA and with the antigenic composition of the vaccines administered during the study period, enabling a standardized assessment of vaccine-elicited functional humoral responses across participants despite heterogeneity in infecting variants. Because Delta/Omicron, particularly Omicron, display substantial antigenic drift and antibody escape, the IC50 values obtained here should be interpreted as cross-reactive/vaccine-antigen-matched functional activity rather than direct neutralization of the infecting variant spike. Even so, the association between higher neutralizing capacity and survival suggests that functional antibody activity may provide information not captured by total binding antibody levels alone. This interpretation is consistent with studies showing that neutralizing activity is an important correlate of protection.^6^^,^^7^^,^^21^^,^^22^

The observation that some deceased patients had high anti-RBD titers but low neutralization capacity suggests that binding antibody quantity alone did not capture protective functionality in these patients. This pattern may reflect qualitative differences in the antibody response, including epitope targeting (neutralizing vs non-neutralizing regions), antibody affinity/avidity and maturation state, and Fc-dependent effector functions that can contribute to viral clearance independently of in vitro neutralization.^23^ Because our study did not directly evaluate Fc-mediated functions or antibody fine specificity, these mechanisms remain hypotheses that warrant investigation in future studies incorporating systems serology, functional Fc assays and variant-matched neutralization readouts. Together, these observations reinforce that antibody quality, not simply total binding levels, may be important for effective immune control and recovery.^22^^,^^24^

Vaccination status may also have contributed to these immunological patterns. The higher proportion of fully vaccinated individuals among recovered patients suggests that booster-induced recall of high-affinity memory B-cells may have contributed to higher neutralization potency. This is consistent with evidence that booster vaccination expands memory B-cells with improved affinity maturation and breadth.^24^ However, because vaccination status, age, comorbidities, disease stage at admission, and other unmeasured variables may be interrelated, the observed association between vaccination and recovery cannot be interpreted causally in this study.

Taken together, the present findings should be interpreted in the context of the limited cohort size, observational design, single admission sampling, and use of an ancestral-spike pseudovirus assay. Although the ancestral-spike design enabled direct antigenic comparison between binding and neutralization measurements, it cannot determine whether these sera neutralized the infecting Delta or Omicron variants with similar potency. Nevertheless, the observation that recovered patients showed higher ancestral-spike neutralizing activity despite comparable total anti-RBD IgG levels provides a biologically consistent basis for future studies using larger longitudinal cohorts, variant-matched pseudovirus assays, and serial measurements of RNAemia, cytokines, and antibody functionality.

In conclusion, our findings underscore the complexity of SARS-CoV-2 pathogenesis and the multifactorial nature of severe COVID-19. In this exploratory cohort, RNAemia was associated with ICU-level disease severity, and higher ancestral-spike neutralizing activity was associated with recovery despite comparable anti-RBD IgG levels. Cytokine levels were higher in hospitalized patients than in individuals with moderate COVID-19 but did not distinguish recovered from deceased patients at a single admission time point. These findings support the biological relevance of systemic viral RNA detection and functional humoral immunity in severe COVID-19, but they require confirmation in larger, longitudinal, variant-matched studies before clinical predictive use can be proposed.

## Declaration of generative AI and AI-assisted technologies in the manuscript preparation process

During the preparation of this work, the authors used ChatGPT (OpenAI) to assist with grammar, spelling, and language editing. After using this tool, the authors reviewed and edited the content as needed and take full responsibility for the content of the submitted manuscript.

## Funding

This work was supported by Fundação de Amparo à Pesquisa do Estado de São Paulo (FAPESP) 20/10127–1 and 2021/11944–6.

## CRediT authorship contribution statement

**Geraldo S. Magalhães:** Conceptualization, Methodology, Formal analysis, Writing – original draft, Writing – review & editing, Supervision. **Sarah N.C. Gimenes:** Investigation, Data curation, Formal analysis, Writing – review & editing. **Bianca C.L.F. Távora:** Investigation. **José A. Portes Junior:** Investigation. **Paula A.L. Calabria:** Investigation. **Leonel I. Montealegre-Sanchez:** Investigation, Data curation, Formal analysis. **Sandra C. Sampaio Vessoni:** Funding acquisition. **Eduardo Massad:** Methodology. **Celina A. Lamas:** Resources. **Sergio S. Muller:** Investigation. **Tania Regina Tozetto-Mendoza:** Resources, Investigation. **Maria Cássia Mendes-Correa:** Resources, Investigation. **Carlos R.R. Carvalho:** Resources, Supervision, Writing – review & editing. **Eliana L. Faquim-Mauro:** Conceptualization, Methodology, Writing – review & editing, Supervision.

## Conflicts of interest

The authors declare no conflicts of interest.

## Data Availability

The datasets generated and/or analyzed during the current study are not publicly available due to patient confidentiality but are available from the corresponding author upon reasonable request.
